# Recovery from Adolescent Anorexia Nervosa and Associations with Diurnal Patterns of Salivary Stress Hormones: A Case Report

**DOI:** 10.1155/2012/798512

**Published:** 2012-12-11

**Authors:** Andrea Oskis, Catherine Loveday, Frank Hucklebridge, David Wood, Angela Clow

**Affiliations:** ^1^School of Psychology, Social Care & Human Sciences, University of West London, Paragon House, Boston Manor Road, Middlesex TW8 9GA, UK; ^2^Department of Psychology, University of Westminster, 309 Regent Street, London W1B 2UW, UK; ^3^School of Life Sciences, University of Westminster, 115 New Cavendish Street, London W1W 6UW, UK; ^4^The Fitzrovia Group Analytic Practice, 51 Warren Street, London W1T 5JH, UK

## Abstract

In the neurodevelopment of adolescent anorexia nervosa (AN), dysregulation of the hypothalamic-pituitary-adrenal (HPA) axis is proposed to be a central component. Furthermore, a therapeutic milieu focusing on affect regulation can contribute much to treatment, given the emotional processing difficulties associated with this disorder. Studies of HPA axis function following such specialist treatments for adolescent AN, however, are rare. This study describes the diurnal pattern of HPA axis activation, including the cortisol awakening response (CAR), in a 16-year-old female diagnosed with AN both during illness and at clinical recovery following milieu therapy with a focus on affect regulation. Specialised single-case study statistics were used to assess whether the patient's data were significantly different from the healthy “norm” at illness and recovery. During illness, her measure of affective problems was outside of the normal range and cortisol and DHEA secretory profiles were significantly elevated across the diurnal period. However, at recovery both her affective state and HPA axis function became comparable to healthy controls. This case study suggests that salivary markers of HPA axis function can be feasibly incorporated into the clinical regime within a specialist adolescent AN residential service and could be used by clinicians to monitor prognosis and interventions.

## 1. Introduction

The neuropathophysiology of anorexia nervosa (AN) remains complex, enigmatic, and controversial, and the disorder is reported to affect 0.5% of Western young women [1]. Experienced clinicians recognise that the treatment of severe, life threatening AN necessitates a multifaceted approach, which best takes place within a specialised eating disorder inpatient unit that can appropriately meet these patients' complex psychosocial and biological needs [2]. For more than 50 years the World Health Organisation has recognised that “the creation of the atmosphere of a therapeutic (milieu) is in itself, one of the most important types of treatment which the hospital can provide” [3]. Milieu therapy, by definition, offers the safety, structure, support, validation, and involvement of living in a therapeutic community [4]. Such a therapeutic environment may hold especial promise for young patients with AN, who are often observed to have great difficulty in regulating and expressing their emotional states. This has led to the suggestion that AN, especially in young girls is “an illness of communication between the world inside themselves and the world outside—an illness of emotional communication” [5]. Research consistently presents findings of significantly greater difficulties in emotional recognition, description, and regulation in females with AN, especially adolescents [6–8].

Of particular importance is that female adolescents with AN have been shown to have a discrepancy between neuroendocrine stress responses, in terms of the hormone cortisol, and *self-reported* emotional arousal to psychosocial stress [9]. This suggests that cortisol could be a useful biomarker for adolescent AN. Indeed, the hypothalamic-pituitary-adrenal (HPA) axis has been proposed as a central component of dysregulation in adolescent AN [10], as it provides an insight into “upstream” influences regarding stress and the effects on health and disease. It has been suggested that an accurate understanding of HPA axis function at different stages of AN would enable more accurate and objective monitoring of therapeutic progress, as well as vulnerability [11].

Both cortisol and dehydroepiandrosterone (DHEA) are important physiological mediators of the HPA axis, and are measureable in saliva. Salivary measures of adrenal steroids are well-established in psychoneuroendocrine research, namely because the collection of samples is non-invasive, thus multiple samples can be performed in the naturalistic environment without the need for medical personnel [12, 13]. Cortisol and DHEA are valid candidates for clinically relevant research because of their distinct rhythmicity; both exhibit a marked circadian rhythm, with a diurnal decline from a morning peak to an evening nadir [14, 15]. Therefore deviations in the “norm” of these rhythms may provide vital insights for understanding different psychopathologies [16, 17]. Awakening is a critical part of the diurnal cycle and cortisol concentrations change rapidly during the immediate post-awakening period, with up to 100% increase [18]. This phenomenon is known as the cortisol awakening response (CAR). The CAR is recognised as a distinct component within the diurnal cortisol rhythm [12], and accordingly should be analysed separately compared to the rest of the daytime profile [19]. Dysregulation in the CAR has been linked with chronic stress, major depressive disorder, and a range of health conditions in adults [12, 20] as well as children and adolescents [21–23].

However, despite the fact that the measurement of salivary HPA axis hormones has been widely used in paediatric research for more than 20 years [24], the employment of such biomarkers in the field of AN, and adolescent AN in particular, is somewhat lacking. The CAR, but not the rest of the diurnal cycle, has recently been examined in a cross-sectional study of adult female outpatients [25], and short day-profiles only have been examined in female adolescents [26]. It remains unknown whether the hypersecretion in AN patients reported by both of these studies is accompanied by normalisation on recovery. Studies of cortisol and DHEA in AN include single, timed blood or saliva samples which only provide snapshots of HPA axis activity (e.g., [27]). A study examining HPA axis function synchronised to individual awakening (to include the CAR) during illness in adolescent AN and at clinical recovery, taking into account a measure of affect, is warranted.

We used a case study longitudinal approach to examine the diurnal pattern of cortisol and DHEA secretion in a single female adolescent both during illness and one year following discharge from treatment (when she was clinically recovered and back in community life). We set out to use the best-recommended methodology for profiling salivary indicators of HPA axis function: multiple samples over the day (separate analysis of the awakening and daytime periods), strict reference to time of awakening, two consecutive sampling weekdays to check for consistency, and careful checks on participant adherence to protocol. Furthermore, the present study adds the complexity of specialised case study statistical analyses to provide additional scrutiny in examining outcomes. Overall, we aimed to examine the effect of milieu therapy focused on affect regulation within a specialised inpatient service for adolescent AN. Our hypothesis was that at baseline, the patient's diurnal rhythms of cortisol and DHEA would be dysregulated and these indicators of HPA axis would normalise when she was recovered.

## 2. Case Presentation

### 2.1. Patient Symptoms and Diagnosis

The case concerns a 16-year-old female (referred to as Bella). On admission to the inpatient treatment service Bella was assessed by the consultant psychiatrist. She met DSM-IV diagnostic criteria for AN (restricting type). She exhibited no comorbid depressive or obsessive compulsive disorders and was not taking any prescribed medication. When she participated in the present study, her weight was 52.2 kg with a BMI of 17.0 kg/m^2^. Bella was undergoing a medium to long stay programme at the service, the duration of which is usually between four to nine months. It has been suggested that undertaking a saliva sampling protocol too close to admission may not be favourable because the participant may not have yet adjusted to their new environment, and the stress of the hospital environment may affect cortisol levels [27], which is why Bella did not take part in the study immediately at admission. She was followed up for this research one year after being discharged from inpatient treatment.

### 2.2. Treatment

The inpatient treatment service offers a “therapeutic milieu” which includes highly skilled nursing care and tailored therapeutic approaches for each young person. The centre's philosophy was one that understood AN to be the expression of major difficulties in self-regulation with consequent effects on emotional communication and on physical health.

Bella followed a comprehensive routine and treatment programme, which, in addition to milieu therapy, included weekly individual psychotherapy, family therapy, and group therapy. Problems identifying emotions in oneself and others and handling relationships were targeted within individual, group and family contexts. This supports research which suggests that exploring the function of emotions and practising emotion recognition might be useful treatment targets for AN [28]. Normalising eating behaviour and regaining a healthy weight was an integral part of Bella's treatment programme. Bella's plan set her rate of weight gain to roughly between 0.5 kg and 1 kg per week, and to steady her weight increase, her individual meal plans were adjusted according to her treatment progress.

### 2.3. Participation in the Present Research

The treatment centre participated in an established activeresearch programme, dedicated to investigating aspects of early onset eating disorders with a particular focus on neuroscience and affect regulation. The present study was part of that research programme, and Bella had volunteered to be a participant. Ethical approval for the study was obtained from both the University of Westminster and Barnet and Haringey Local Research Ethics Committees. The protocol involved the following assessments, described below. The questionnaire was chosen to assess Bella's eating pathology pre- and post-participation. After informed written consent was provided by both Bella and her parents, the following assessments were completed.

#### 2.3.1. Eating Disorders Inventory-3 (EDI-3) [29]

The EDI-3 contains 91 items which form various eating disorder risk scales and psychological scales that are rated using a six-point scoring format; the higher the scores for each scale, the more symptomatic the individual. The different scales regarding disordered behaviours and psychological traits can be collapsed to form two overall composite scores, named eating disorder risk and the general psychological maladjustment, the latter of which includes a measure of affective problems.

#### 2.3.2. Salivary Cortisol and DHEA

Bella was provided with a pack containing saliva sampling materials. She was instructed to collect saliva samples at awakening, 15, 30, and 45 minutes and 6 and 12 hours post-awakening on two consecutive weekdays. Saliva was collected by passive drool through a straw into the appropriately labelled small, plastic Eppendorf tube. For at least 30 minutes prior to the collection of each sample, she had to adhere by guidelines of nil by mouth other than water and the avoidance of vigorous exercise and brushing teeth. Other than these requests for compliance, Bella was free to follow her normal daily routine. After collection, Bella's samples were frozen (−20°C). On the last day of Bella's participation, the researcher used insulated packs to transfer samples to the laboratory where they were stored at −20°C until assay. On each study day, Bella recorded her awakening time, method of waking up (whether naturally or by alarm clock) and the exact times of collection of saliva samples. To maximise adherence to protocol, the timing of all Bella's samples was supervised by nursing staff.

In the laboratory, Bella's saliva samples were thawed and centrifuged at 1500 xg (3000 rpm) for 15 minutes. Her cortisol concentration were determined by the Expanded Range High Sensitivity Enzyme Linked Immuno-Sorbent Assay developed by Salimetrics LLC (USA). Similarly, the Salimetrics Salivary DHEA Enzyme. For full details of the assay procedure, see Oskis et al. [15].

### 2.4. Statistical Approach to the Case Report

Researchers now have the opportunity to utilise statistical techniques that allow single case studies to be amenable to the same statistical research questions as larger-*N* research studies. These have been especially useful within neuropsychology, where such research findings translate to the clinical setting and facilitate the development a case's profile of cognitive strengths and weakness [30]. However, these techniques are uncommon within neuroendocrinology where the use of single case studies is rare. Nevertheless, since our endeavour was the same, namely to see whether our case study was significantly different on assessments compared to a matched control sample, we chose to use the approach developed by Crawford and Howell [30]. This method is effectively a modified independent samples *t*-test in which the individual is treated as a sample of *n* = 1. The test is robust in that it controls the Type I error rate regardless of the size of the control sample. More recently, Crawford et al. [31] have further developed this method so that as well as testing for a statistically significant difference, an effect size index for the difference between the case and controls can also be obtained. This index of effect size, termed *z*_CC_, is analogous to Cohen's *d* and is an estimate of the average difference, measured in standard deviation units, between a case's score and the score of a randomly chosen member of the control population. The computer programme Singlims-ES implements these methods and was used to analysis of Bella's assessments compared to a control group.

### 2.5. Outcome

Bella's data were examined against a backdrop of comparator participant data derived from a parallel, larger study investigating HPA axis activity in healthy females [15]. This sample contained 15 post-menarche female adolescents who were age matched (mean (±SD) age 16.67 (±0.49) years), with normal BMI (21.39 kg/m^2^ ± 3.18).

Using Crawford and Howell's [30] approach, Bella's mean cortisol and DHEA variables for the two samplings day were analysed against the corresponding values from the healthy control group to ascertain whether her values were significantly different. Composites were computed to represent the total concentrations and dynamic of each hormone, in line with conventions in neuroendocrine research see [32].

#### 2.5.1. Baseline

**Figure 1:**
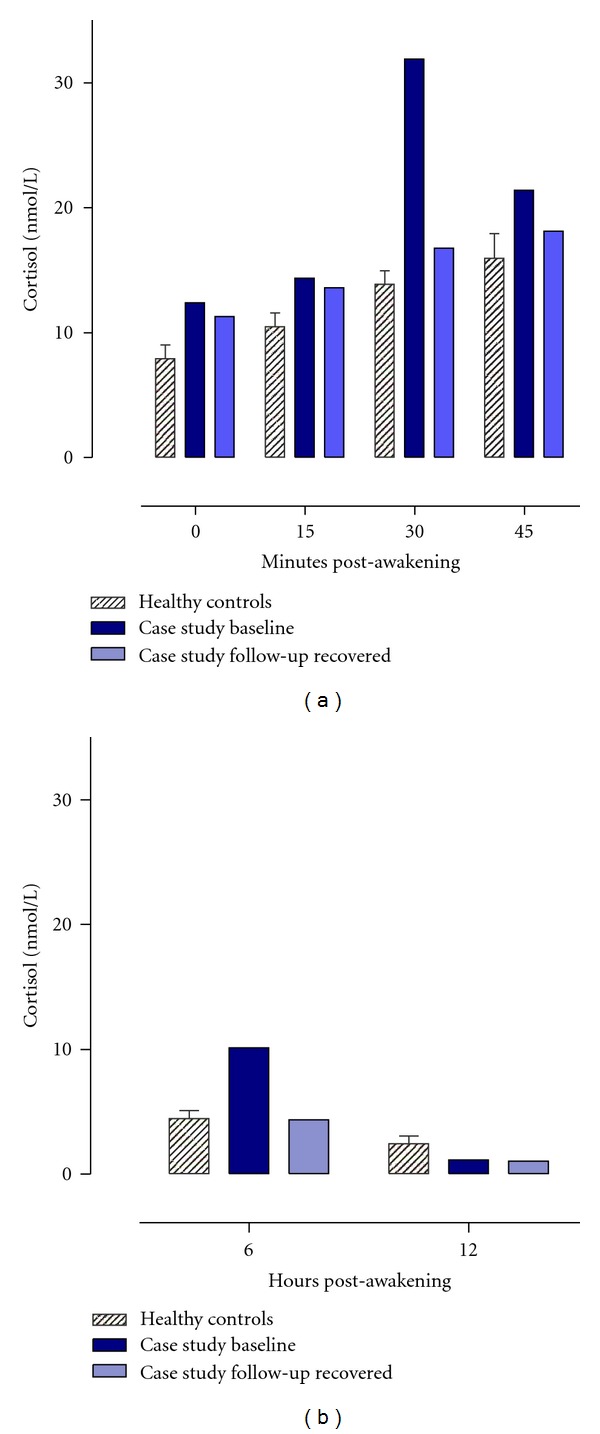
Mean (±SEM) salivary cortisol concentrations (nmol/l) after awakening and over the daytime period for the healthy control group (*n* = 15) and case study Bella at baseline and when recovered at one year follow-up.

Both Table 1 and Figure 1 illustrate Bella's cortisol profile at baseline when she was ill with AN, and one year after being discharged from the inpatient service when she was recovered. At baseline, Bella's overall levels of cortisol after awakening were significantly higher than those of the control group. However, the dynamic of Bella's CAR mean increase did not differ significantly to that of the controls. For cortisol over the rest of the day, once again Bella's overall levels of cortisol from 6 to 12 hours post-awakening were significantly greater but there was no difference in the decline over this daytime period compared to the control group.

**Figure 2:**
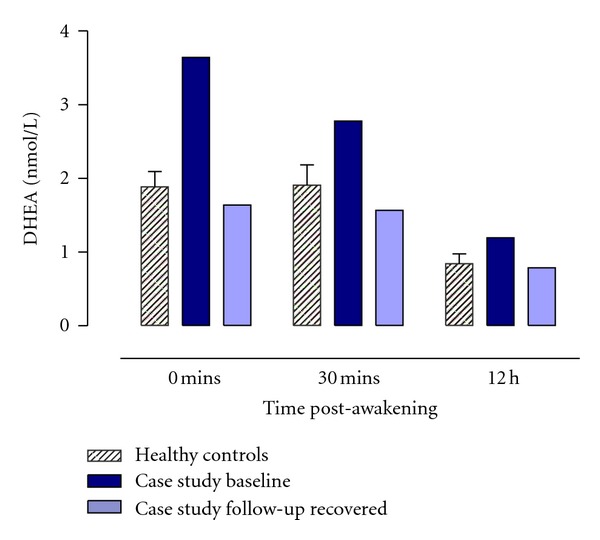
Mean (±SEM) salivary DHEA concentrations (nmol/l) after awakening and over the daytime period for the healthy control group (*n* = 15) and case study Bella at baseline and when recovered at one year follow-up.

Unlike cortisol, DHEA is not characterised by a marked awakening response [14, 15]. Given this, only the samples taken at awakening, 30 minutes, and 12 hours post-awakening were analysed for DHEA. Bella's concentrations in the 30 minute post-awakening period and in the evening were significantly higher than the healthy control group (see Table 1 and Figure 2). As can also be seen in Table 1, Bella's eating pathology assessments at baseline confirmed her diagnosis and her EDI-3 scores for eating disorder risk, particularly the body dissatisfaction scale and general psychological maladjustment, were significantly higher than those of the control group. Bella's score for affective problems was also significantly higher than controls.

#### 2.5.2. Follow-Up

Bella was followed up one year after being discharged from her programme at the service. She was managing and maintaining her weight in the community and at follow-up Bella's weight was 58.5 kg with a BMI of 19 kg/m^2^. After leaving inpatient treatment, Bella was transferred to outpatient support in the form of a community practitioner nurse whom she saw roughly every six weeks. At the time of follow-up participation, Bella was at college getting ready to take her A-level exams in a few months' time and hoping to go to university in the coming year. She was happy socially, and had friends at college, including a boyfriend. She had just also started a part-time job in a local retail establishment.

Bella completed the same protocol as before. Crawford and Howell's [30] approach was used once again to compare Bella's mean cortisol and DHEA composites for the two samplings day to the corresponding values from the healthy control group. One year after being discharged from the inpatient service when she was recovered, Bella's overall levels of cortisol after awakening were not significantly different to the control group and neither were her overall levels for the rest of the day. Furthermore, the dynamic of Bella's cortisol profile did not differ significantly to that of the controls for either the CAR or the rest of the daytime period. Bella's cortisol profiles at follow-up can be seen in Figure 1 and Table 1. Similarly for DHEA, Bella's concentrations were all comparable to the control group for both the 30 minute post-awakening period and also in the evening. Bella's DHEA profiles at follow-up can be seen in Figure 2, and the accompanying statistics can be seen in Table 1.

One year on, all of Bella's EDI-3 scores had lowered and were not significantly different compared to the healthy control group (see Table 1). Her general psychological maladjustment and her affective problems had improved to be comparable to the healthy group, and notably, the EDI-3 qualitative classification for these two indices had lowered from “typical clinical” during illness to “low clinical” at follow-up.

## 3. Discussion

During illness, Bella's measure of affective problems was outside of the normal range and her cortisol and DHEA secretory profiles were significantly elevated across the diurnal period. Despite hypersecretion of both hormones, the dynamics of the diurnal profiles (e.g., for the CAR and diurnal declines) remained comparable to controls, which indicated hyperactivity of HPA axis drive overall, and not CAR specific mechanisms (see [33] for a detailed discussion of these physiological mechanisms). Bella's clinical symptoms and overall levels of cortisol and DHEA were no longer significantly different to controls one year post-discharge from treatment. At this point she was recovered and maintaining life in the community. Bella's EDI measure of her affective problems at follow-up had improved compared to illness phase. This case study adds further support to existing empirical work which illustrates that female adolescents with AN have difficulties with emotional recognition and regulation [6] and that intensive, affect regulation focused therapeutic milieu for the treatment of AN can have a positive outcome.

The present case study is novel in that it illustrates that the statistical techniques commonly used to illuminate clinical outcomes in neuropsychology can be applied to neuroendocrinology. Our approach has demonstrated that research techniques can provide information for clinicians that may potentially be used to monitor each individual's therapeutic progress. These techniques can be used in terms of psychometric assessments such as the EDI, but also for indices of HPA axis function. Although Bella was assessed during her illness/treatment phase and one year post-discharge, the statistical approach would be applicable at any point during treatment and would enable both therapeutic progress and vulnerability to be monitored [11].

In line with the present study, a recent cohort study of 210 females with AN reports that cortisol could be used as an important prognostic predictor [34]. These authors found increased levels of cortisol in AN patients who developed critical states during the three month timescale of their study, however cortisol was measured via plasma. It is noteworthy that venipuncture can significantly enhance cortisol levels in some participants, most probably reflecting a psychological stress response [35]. In another area of adolescent psychopathology however, *salivary* HPA axis rhythms are presently being utilised to monitor intervention efforts for adolescent depression (see [36]). Thus, salivary, as opposed to plasma, indicators of HPA axis function could similarly and feasibly be used to monitor prognosis and intervention for adolescent AN. In line with the methodological approach of the present case study, we propose that collecting saliva by chewing on a cotton swab may be inappropriate and stressful for individuals with AN; drooling passively into a plastic tube, is considered the most non-intrusive and best-tolerated method of saliva sampling in this population [37].

In conclusion, we encourage broader employment of the neuroendocrine and the specialised statistical methods outlined in this case study, as both approaches provide useful information equally for researchers and clinicians working in the field of adolescent AN. Furthermore, our case study of Bella has illuminated the issue of assessing recovery in AN, and supports the idea of clinicians incorporating both measures of affect and salivary HPA axis assessments, from admission to post-treatment. Although our study is limited to a single case study, our endeavour has been to provide preliminary evidence that HPA axis activity can be routinely measured via an easily accessible biological fluid as part of a therapeutic treatment milieu for AN. Based on the results of the current case study, we encourage future work to investigate salivary cortisol and DHEA in larger group designs to determine the extent to which these variables are relevant for AN, especially with regard to measures of affect.

## Figures and Tables

**Table 1:** Healthy control group (*n* = 15) compared to case study Bella at baseline and when recovered at one year follow-up.

	Healthy controls		Bella							
	Mean	SD	Baseline mean	*t*	*P*	*z*_CC_	One year follow-up mean	*t*	*P*	*z*_CC_
Awakening cortisol overall levels (mean in nmol/L)	36.37	(±10.70)	63.19*	2.43	0.02	2.51	45.10	0.80	0.22	0.82
Awakening cortisol dynamic (mean in nmol/L)	5.51	(±4.99)	8.80	0.65	0.26	0.67	5.27	−0.05	0.48	−0.05
Daytime cortisol overall levels (mean in nmol/L)	6.88	(±2.18)	11.30*	2.08	0.02	2.14	5.40	−0.66	0.26	−0.68
Daytime cortisol dynamic	0.69	(±1.32)	2.11	1.06	0.15	1.09	3.00	1.69	0.11	1.75
DHEA awakening levels (mean in nmol/L)	1.90	(±0.81)	3.64*	2.09	0.03	2.15	1.64	−0.31	0.38	−0.32
DHEA evening levels (mean in nmol/L)	0.53	(±0.30)	1.20*	2.07	0.03	2.17	0.78	0.10	0.46	−0.10
EDI-3 eating disorder risk	28.67	(±6.78)	40.00*	1.62	0.01	1.67	35.00	−1.16	0.13	1.20
EDI-3 drive for thinness	26.07	(±8.29)	38.00	1.39	0.09	1.44	32.00	0.69	0.50	0.72
EDI-3 bulimia	42.73	(±5.16)	41.00	−0.33	0.75	−0.34	41.00	−0.33	0.75	−0.34
EDI-3 body dissatisfaction	31.00	(±6.51)	48.00*	2.53	0.01	2.62	43.00	1.79	0.10	1.84
EDI-3 general psychological maladjustment	36.87	(±5.91)	49.00*	1.99	0.03	2.05	35.00	−0.31	0.38	−0.32
EDI-3 affective problems	39.82	(±4.45)	50.00*	2.18	0.03	2.27	35.00	1.09	0.30	1.14

^∗^ *P* < 0.05 (indicates statistically significant difference to corresponding control value).
